# Gamma Interferon Mediates Experimental Cerebral Malaria by Signaling within Both the Hematopoietic and Nonhematopoietic Compartments

**DOI:** 10.1128/IAI.01035-16

**Published:** 2017-10-18

**Authors:** Ana Villegas-Mendez, Patrick Strangward, Tovah N. Shaw, Ivana Rajkovic, Vinko Tosevski, Ruth Forman, Werner Muller, Kevin N. Couper

**Affiliations:** a Faculty of Biology, Medicine and Health, University of Manchester, Manchester, United Kingdom; b Institute of Experimental Immunology, Inflammation Research Unit, University of Zurich, Zurich, Switzerland; University of South Florida

**Keywords:** malaria, IFN-γ, cerebral malaria, brain, pathology, immunopathology

## Abstract

Experimental cerebral malaria (ECM) is a gamma interferon (IFN-γ)-dependent syndrome. However, whether IFN-γ promotes ECM through direct and synergistic targeting of multiple cell populations or by acting primarily on a specific responsive cell type is currently unknown. Here, using a panel of cell- and compartment-specific IFN-γ receptor 2 (IFN-γR2)-deficient mice, we show that IFN-γ causes ECM by signaling within both the hematopoietic and nonhematopoietic compartments. Mechanistically, hematopoietic and nonhematopoietic compartment-specific IFN-γR signaling exerts additive effects in orchestrating intracerebral inflammation, leading to the development of ECM. Surprisingly, mice with specific deletion of IFN-γR2 expression on myeloid cells, T cells, or neurons were completely susceptible to terminal ECM. Utilizing a reductionist *in vitro* system, we show that synergistic IFN-γ and tumor necrosis factor (TNF) stimulation promotes strong activation of brain blood vessel endothelial cells. Combined, our data show that within the hematopoietic compartment, IFN-γ causes ECM by acting redundantly or by targeting non-T cell or non-myeloid cell populations. Within the nonhematopoietic compartment, brain endothelial cells, but not neurons, may be the major target of IFN-γ leading to ECM development. Collectively, our data provide information on how IFN-γ mediates the development of cerebral pathology during malaria infection.

## INTRODUCTION

Plasmodium berghei ANKA infection of susceptible strains of mice leads to the development of a fatal cerebral pathology, termed experimental cerebral malaria (ECM). Despite some debate, this murine infection model represents the best and most widely utilized animal model for human cerebral malaria (HCM), which is the major cause of morbidity and mortality of humans infected with Plasmodium falciparum (1–4). Although the pathogenesis of ECM is still not fully understood, it is well established that gamma interferon (IFN-γ) plays a central role in the development of the condition (5). Thus, IFN-γ- and IFN-γ receptor (IFN-γR)-deficient mice on susceptible backgrounds are fully protected against the development of cerebral pathology (6–8). The resistance of IFN-γR-deficient animals is associated with attenuated parasite accumulation in the brain (9, 10), reduced migration (and/or retention) of leukocytes within the brain (7, 11), decreased chemokine expression in the brain (7, 12), lowered activation of cerebral microvessels (6, 7), and decreased cross-presentation of parasite antigens by cerebral endothelial cells (13). We have shown that IFN-γ production solely by CD4^+^ T cells is sufficient to cause ECM during P. berghei ANKA infection (14). However, the cell populations directly targeted by IFN-γ during P. berghei ANKA infection, thus promoting the development of ECM, are presently unknown.

The functional IFN-γR, a heterodimeric complex composed of the IFN-γ receptor 1 (IFN-γR1) and IFN-γR2 chains, can be expressed on many different cell types (15, 16). IFN-γR1, the major ligand binding subunit, is ubiquitously expressed, whereas the expression of the nonbinding, signal-transducing IFN-γR2 subunit is generally low and is tightly controlled (15, 16). Consequently, IFN-γR2 expression, rather than IFN-γR1 expression, controls the responsiveness of cells to IFN-γ (15, 17). In nonmalaria models, it has been shown that IFN-γ enhances antigen processing, major histocompatibility complex (MHC) and costimulatory marker expression, and cytokine production in dendritic cells and macrophages/monocytes (15, 16). IFN-γ can also act on T cells, orchestrating CD4^+^ T cell and CD8^+^ T cell activation and differentiation, as well as apoptosis (18–24). Moreover, IFN-γ can directly modify the function and status of brain-resident and -specialized cell populations, including neurons, brain endothelial cells, microglial cells, and astrocytes, in a variety of inflammatory settings, including malaria (25–30). Combined, these observations indicate that IFN-γ may mediate ECM development by targeting a specific cell type, in a particular location, at a precise time of infection. Alternatively, it may cause cerebral pathology during malaria infection by functioning within a complex cellular network, acting synergistically on different cell types.

In this study, we have investigated the cell population(s) and compartments that IFN-γR signals within to mediate ECM development during P. berghei ANKA infection. Utilizing novel cell- and compartment-specific IFN-γR2-deficient mice (31), we demonstrate that IFN-γ causes ECM by signaling within both the hematopoietic and nonhematopoietic compartments. Within the brain, IFN-γR signaling within the two compartments was additive, which led to severe neuroinflammation. Within the hematopoietic and nonhematopoietic compartments, IFN-γR2 expression by myeloid cells, T cells, and neurons was, individually, not required for the development of ECM. Importantly, we show that brain endothelial cells were highly responsive to IFN-γ in combination with tumor necrosis factor (TNF). Thus, within the nonhematopoietic compartment, IFN-γ may mediate ECM by directly targeting brain endothelial cells. The results in this study improve our knowledge of the IFN-γR-expressing cell populations that may contribute to the IFN-γ-dependent development of cerebral pathology during malaria infection.

## RESULTS

### IFN-γ promotes the development of ECM by signaling within both the hematopoietic and nonhematopoietic compartments.

**FIG 1:**
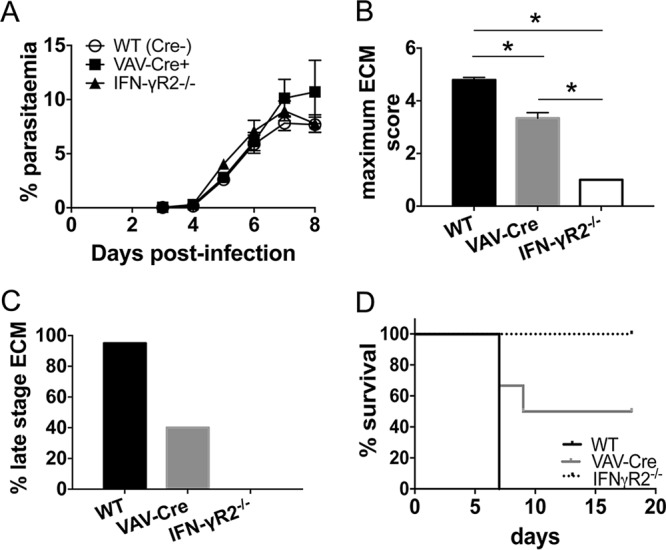
Mice lacking IFN-γR signaling within the hematopoietic compartment display intermediate susceptibility to ECM. WT, IFN-γR^−/−^, and VAV-Cre^+^ IFN-γR2^flox/flox^ mice were infected intravenously (i.v.) with 10^4^ P. berghei ANKA-parasitized red blood cells (pRBCs). (A, B) The course of infection was monitored by assessing peripheral parasite levels (A) and the maximum ECM score (B) observed during the course of the experiment. Results are the combined mean values ± standard errors of the means (SEM) for the groups from six independent experiments, with 3 to 7 mice per group per experiment. (C) Percentage of mice of each strain within all experiments that developed late-stage ECM (score of ≥4) during the course of infection. Total numbers of mice used in experiments were as follows: WT, *n* = 43; VAV-Cre^+^ IFN-γR2^flox/flox^, *n* = 32; IFN-γR^−/−^, *n* = 39. (D) Survival of the different strains. Results are representative of 2 independent experiments with 4 or 5 mice per group. *, *P* < 0.05 between defined groups. Statistical significance was tested using Kruskal-Wallis test with Tukey's *post hoc* test.

To identify the cellular compartments that IFN-γR signals within to mediate the development of ECM, we infected VAV-Cre^+^ IFN-γR2^flox/flox^ mice (which lack IFN-γR2 expression on all hematopoietic-origin cells) with P. berghei ANKA and compared their susceptibility to ECM with the susceptibilities of globally IFN-γR2^−/−^ mice and wild-type (WT; VAV-Cre^−^ IFN-γR2^flox/flox^ littermate) mice. WT, globally IFN-γR2^−/−^, and VAV-Cre^+^ IFN-γR2^flox/flox^ mice developed comparable peripheral parasite burdens, indicating that neither global nor hematopoietic cell-specific IFN-γR expression contributes to peripheral parasite control during the early phases of P. berghei ANKA infection (Fig. 1A). As expected (6–8, 11), globally IFN-γR2^−/−^ mice were completely resistant to the development of ECM (Fig. 1B to D), whereas almost 100% of WT mice developed signs of late-stage ECM, typically on day 7 of infection (Fig. 1C and D). In contrast, VAV-Cre^+^ IFN-γR2^flox/flox^ mice displayed intermediate resistance to ECM: all VAV-Cre^+^ IFN-γR2^flox/flox^ mice developed prodromal signs of ECM (mean score of 3.34 on the grading system) (Fig. 1B), but 60% of the total number of mice used in the study (19/32) were protected from late-stage terminal ECM (Fig. 1C), and in the experiments where survival was specifically assessed, 55% of the VAV-Cre^+^ IFN-γR2^flox/flox^ mice (6/11) survived the window period of ECM development (days 6 to 12 postinfection) (Fig. 1D). Thus, both hematopoietic and nonhematopoietic IFN-γR signaling contribute toward robust development of terminal late-stage ECM during P. berghei ANKA infection.

### IFN-γR signaling within the hematopoietic compartment does not strongly influence splenic immunity during infection.

**FIG 2:**
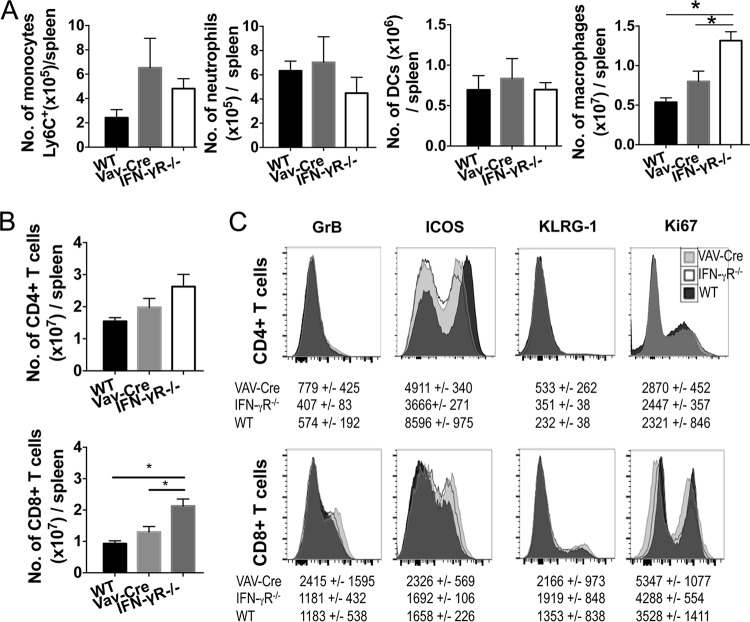
IFN-γR signaling within the hematopoietic compartment does not substantially influence splenic immune responses during P. berghei ANKA infection. WT, IFN-γR2^−/−^, and VAV-Cre^+^ IFN-γR2^flox/flox^ mice were infected i.v. with 10^4^ P. berghei ANKA pRBCs. (A to C) On day 7 of infection, when WT mice developed late-stage ECM, spleens were removed and analyzed by flow cytometry. (A) Numbers of inflammatory monocytes, neutrophils, dendritic cells (DCs), and F4/80^+^ macrophages in spleens. (B, C) Numbers (B) and representative activation (C) of splenic CD4^+^ T cells and CD8^+^ T cells on day 7 of infection. Results in panels A and B are the mean value ± SEM for each group with 3 to 6 mice per group. Results represent data from 2 independent experiments. *, *P* < 0.05 between defined groups. Statistical significance tested using one-way ANOVA with Tukey's *post hoc* test. (C) Representative histograms and associated mean values and standard deviations (SD) of the groups, with total numbers of 7 to 11 for each group combined from 2 independent experiments.

We assessed whether abrogated IFN-γR2 signaling within the hematopoietic compartment modulated the generation and/or maintenance of innate and adaptive splenic immune responses during infection, contributing to the intermediate resistance of VAV-Cre^+^ IFN-γR2^flox/flox^ mice to ECM. Within the innate compartment, the numbers of inflammatory monocytes, neutrophils, and classical dendritic cells (CD11b^+^ and CD8^+^) were unaltered in the spleens of IFN-γR^−/−^ and VAV-Cre^+^ IFN-γR2^flox/flox^ mice compared with the numbers in WT mice on day 7 of infection (Fig. 2A). In contrast, the numbers of F4/80^+^ macrophages were significantly increased in the spleens of IFN-γR^−/−^ mice compared with the numbers in VAV-Cre^+^ IFN-γR2^flox/flox^ mice and WT mice on day 7 of infection (Fig. 2A). Thus, nonhematopoietic and/or any global IFN-γR2 signaling appears to specifically limit splenic macrophage numbers during P. berghei ANKA infection.

There was a trend toward higher numbers of splenic CD4^+^ T cells in IFN-γR^−/−^ mice but not in VAV-Cre^+^ IFN-γR2^flox/flox^ mice compared with the numbers in WT mice on day 7 of infection (Fig. 2B). Similarly, splenic CD8^+^ T cell numbers were significantly increased in IFN-γR^−/−^ mice but not VAV-Cre^+^ IFN-γR2^flox/flox^ mice compared with the numbers in WT mice on day 7 of infection (Fig. 2B). Splenic CD4^+^ T cell and CD8^+^ T cell activation was, however, largely unaltered, as determined by comparing the levels of granzyme B, KLRG-1, and Ki-67 expression in IFN-γR^−/−^ mice to the levels in VAV-Cre^+^ IFN-γR2^flox/flox^ mice and WT mice on day 7 of infection (Fig. 2C). Of the markers examined, only ICOS was differentially expressed by splenic CD4^+^ T cells, but not CD8^+^ T cells, in IFN-γR^−/−^ mice compared with VAV-Cre^+^ IFN-γR2^flox/flox^ mice and WT mice (Fig. 2C). Combined, these data suggest that hematopoietic IFN-γR signaling does not substantially control the splenic immune response during P. berghei ANKA infection; however, nonhematopoietic and/or synergistic global IFN-γR signaling may affect the numbers but not the general activation of specific splenic innate and adaptive immune cell populations, most probably through promoting cellular apoptosis rather than controlling cellular migration to diverse nonlymphoid tissues (11).

### IFN-γR signaling within the hematopoietic and nonhematopoietic compartments synergistically defines the level of cerebral inflammation during infection.

**FIG 3:**
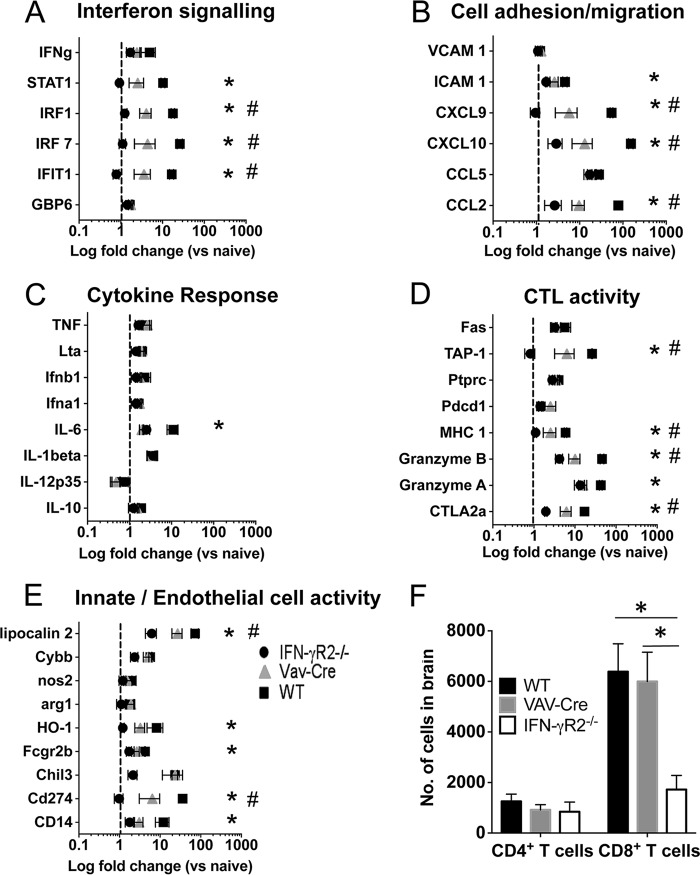
IFN-γR signals synergistically within nonhematopoietic and hematopoietic compartments to promote intracerebral inflammation during P. berghei ANKA infection. WT, IFN-γR^−/−^, and VAV-Cre^+^ IFN-γR2^flox/flox^ mice were infected i.v. with 10^4^ P. berghei ANKA pRBCs. (A to E) On day 7 of infection, brains were removed and expression of selected genes in whole brains was determined by NanoString analysis. Selected genes were grouped according to functional activity, including interferon signaling (A), cell adhesion/migration (B), cytokine response (C), CTL activity (D), and innate/endothelial cell activity (E). Results are presented as log fold change in gene expression compared with mean gene expression in naive brains (with dotted lines representing equivalent magnitude of gene expression as in naive brains). (F) On day 7 of infection, brains were removed and the numbers of CD4^+^ T cells and CD8^+^ T cells were quantified. Results in all panels are the mean values ± SEM of the groups, with 4 to 8 brains per group. For the data shown in panels A to E, brains were obtained from 2 independent experiments. Results in panel F are representative of two independent experiments. *, *P* < 0.05 for WT versus IFN-γR2^−/−^ mice; #, *P* < 0.05 for WT versus VAV-Cre mice. Statistical significance was tested using one-way ANOVA with Tukey's *post hoc* test.

Next, we utilized NanoString gene expression analysis to examine how hematopoietic and nonhematopoietic compartment-specific IFN-γR signaling coordinately shape the inflammatory landscape within the brain during P. berghei ANKA infection, promoting ECM development. As expected, the levels of expression of a number of genes associated with interferon signaling, cell adhesion/migration, cytotoxic T lymphocyte (CTL) activity, and innate/endothelial cell activity were significantly elevated within the brains of WT mice experiencing ECM compared with their expression in the brains of ECM-resistant IFN-γR^−/−^ mice (Fig. 3A to E). Notably, the expression of key genes, including those encoding CXCL9, CXCL10, granzyme B, and TAP-1, all of which have been shown to be involved in ECM pathogenesis (32), was dampened rather than completely abrogated in the brains of infected VAV-Cre^+^ IFN-γR2^flox/flox^ mice compared with their expression in brains from WT mice (Fig. 3B and D). Indeed, for all genes identified as differentially expressed in the brains of infected WT mice compared with their expression in infected IFN-γR^−/−^ mice, including those encoding the IFN signaling molecules STAT1, IRF1, IRF7, and IFIT1, intermediate gene expression was observed in the brains of VAV-Cre^+^ IFN-γR2^flox/flox^ mice (Fig. 3A to E). Interestingly, although the numbers of intracerebral CD8^+^ T cells were significantly decreased in infected globally IFN-γR2^−/−^ mice compared with the numbers in WT mice on day 7 of infection, potentially contributing to the reduced inflammatory landscape in the brains of globally IFN-γR2^−/−^ mice, the numbers of intracerebral CD8^+^ T cells were comparable in infected VAV-Cre^+^ IFN-γR2^flox/flox^ mice and WT mice (Fig. 3F). Consequently, these results suggest that ablation of IFN-γR signaling within the hematopoietic compartment in VAV-Cre^+^ IFN-γR2^flox/flox^ mice led to a generalized reduction of inflammation within the brain during infection, rather than the suppression of individual or specific modules of genes. However, the ameliorated cerebral inflammation in VAV-Cre^+^ IFN-γR2^flox/flox^ mice was not simply due to reduced CD8^+^ T cell accumulation in the brain.

### IFN-γR2 signaling in peripheral myeloid cells and T cells is dispensable for ECM development.

**FIG 4:**
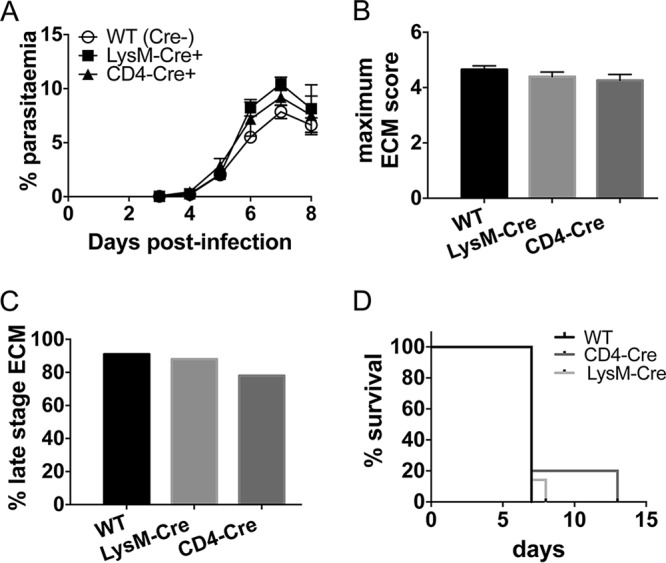
IFN-γR signaling within LysM- and CD4-expressing cells is redundant for ECM development. WT, LysM-Cre^+^ IFN-γR2^flox/flox^, and CD4-Cre^+^ IFN-γR2^flox/flox^ mice were infected i.v. with 10^4^ P. berghei ANKA pRBCs. (A, B) Course of infection was monitored by assessing peripheral parasite levels (A) and the maximum ECM score observed during the course of the experiment (B). Results are the combined mean values ± SEM of the groups from five independent experiments, with 3 to 7 mice per group per experiment. (C) Percentage of mice of each strain within all experiments that developed late-stage ECM (score of ≥4) during the course of infection. Total numbers of mice used in experiments were as follows: WT, *n* = 23; LysM-Cre^+^ IFN-γR2^flox/flox^, *n* = 24; CD4-Cre^+^ IFN-γR2^flox/flox^, *n* = 23. (D) Survival of the different strains. Results are representative of 2 independent experiments with 4 or 5 mice per group.

To examine which cell populations respond to IFN-γ within the hematopoietic compartment to mediate IFN-γ-dependent ECM development, we generated CD4-Cre^+^ IFN-γR2^flox/flox^ mice (which lack IFN-γR2 expression specifically on CD4^+^ and CD8^+^ T cells) and LysM-Cre^+^ IFN-γR2^flox/flox^ mice (which lack IFN-γR2 expression specifically on Ly6C^+^ and Ly6C^−^ monocytes, neutrophils, and most mature macrophage populations). We utilized these mice because, although the splenic T cell and myeloid cell responses were not dramatically attenuated in infected VAV-Cre^+^ IFN-γR2^flox/flox^ mice (Fig. 2), it was possible that cell-specific IFN-γR2 signaling controlled T cell and myeloid responses in the brain and other nonlymphoid compartments, influencing the level of neuroinflammation (Fig. 3) and development of ECM. Indeed, we have previously shown that the activation of intracerebral CD4^+^ T cells is reduced in P. berghei ANKA-infected IFN-γ^−/−^ mice (11). As expected, CD4-Cre^+^ IFN-γR2^flox/flox^ and LysM-Cre^+^ IFN-γR2^flox/flox^ mice developed peripheral parasite burdens similar to those in littermate control (Cre^−^) mice (Fig. 4A). However, importantly, CD4-Cre^+^ IFN-γR2^flox/flox^ and LysM-Cre^+^ IFN-γR2^flox/flox^ mice were as susceptible to ECM as littermate control mice, exhibiting mean ECM scores equivalent to those of WT mice (Fig. 4B). Moreover, CD4-Cre^+^ IFN-γR2^flox/flox^ and LysM-Cre^+^ IFN-γR2^flox/flox^ mice developed late-stage ECM with frequencies comparable to that of WT mice (total of 18/23, 21/24, and 21/23, respectively) (Fig. 4C). In experiments where survival was specifically assessed, infected CD4-Cre^+^ IFN-γR2^flox/flox^ and LysM-Cre^+^ IFN-γR2^flox/flox^ mice also succumbed to ECM with frequencies similar to that of WT mice (total of 9/11, 10/11, and 11/11) (Fig. 4D). Consequently, these results demonstrate that the intermediate resistance of VAV-Cre^+^ IFN-γR2^flox/flox^ mice was not due to the specific and individual loss of IFN-γR signaling within the T cell or myeloid lineages.

### ECM can develop in the absence of IFN-γR2 signaling in neurons and astrocytes.

**FIG 5:**
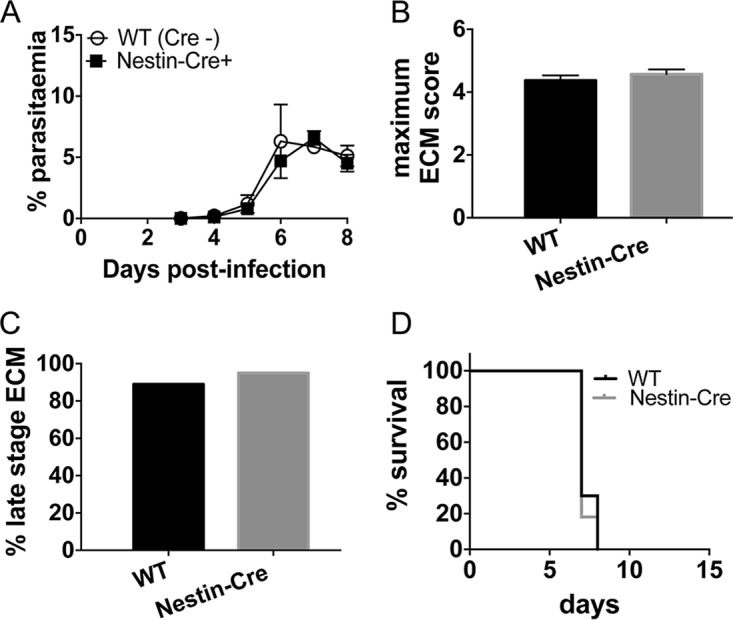
IFN-γR signaling within Nestin-expressing cells is redundant for ECM development. WT and Nestin-Cre^+^ IFN-γR2^flox/flox^ mice were infected i.v. with 10^4^ P. berghei ANKA pRBCs. (A, B) The course of infection was monitored by assessing peripheral parasite levels (A) and the maximum ECM score observed during the course of the experiment (B). Results are the combined mean values ± SEM of the groups from three independent experiments, with 3 to 10 mice per group. (C) Percentage of mice of each strain within all experiments that developed late-stage ECM (score of ≥4) during the course of infection. Total numbers of mice used within experiments were as follows: WT, *n* = 28; Nestin-Cre^+^ IFN-γR2^flox/flox^, *n* = 20. (D) Survival of the different strains. Results are representative of 2 independent experiments with 4 or 5 mice per group.

The VAV-Cre^+^ IFN-γR2^flox/flox^ mouse experimental data highlighted an important role for IFN-γR expression by nonhematopoietic cells in promoting terminal ECM development. Neurons express IFN-γR, and IFN-γ can directly induce neuronal damage (25). Neuronal damage has also been reported during P. berghei ANKA infection (33). Thus, to investigate whether IFN-γR signaling within neurons contributes to ECM development, we generated Nestin-Cre^+^ IFN-γR2^flox/flox^ mice, as Nestin-Cre has previously been shown to drive strong Cre-mediated gene recombination in neurons (34). Notably, Nestin-Cre also promotes efficient gene recombination in astrocytes (35), which can be direct target cells of IFN-γ (5, 26, 28, 29), allowing us to use the Nestin-Cre^+^ IFN-γR2^flox/flox^ mice to also assess the specific role of IFN-γR signaling in glial cells in the development of ECM. Nestin-Cre^+^ IFN-γR2^flox/flox^ mice developed peripheral parasite burdens equivalent to those in littermate control mice (Fig. 5A). In contrast to our expectations, Nestin-Cre^+^ IFN-γR2^flox/flox^ mice were as susceptible to ECM during P. berghei ANKA infection as littermate Cre^−^ control mice, with all Nestin-Cre^+^ IFN-γR2^flox/flox^ mice developing late-stage ECM (20/20) and, in experiments where survival was specifically assessed, succumbing to infection on day 7 of infection (10/10) (Fig. 5B to D).

### IFN-γ synergizes with TNF to activate brain endothelial cells *in vitro*.

**FIG 6:**
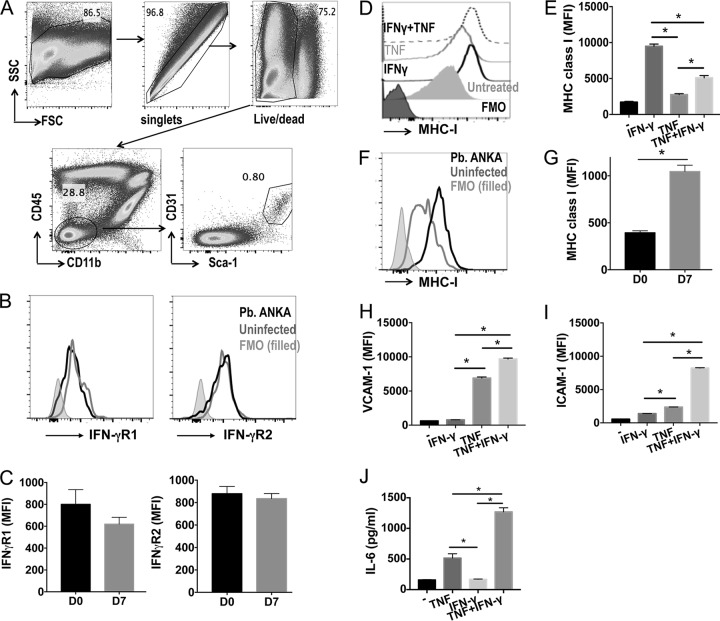
IFN-γ promotes direct activation of brain endothelial cells under inflammatory conditions. WT mice were infected i.v. with 10^4^ P. berghei ANKA pRBCs. Brains were removed from infected mice on day 7 of infection and from naive mice. (A to C) Representative plots showing gating strategy to identify brain endothelial cells (A), representative histograms (B), and mean fluorescence intensities (MFI) of IFN-γR1 and IFN-γR2 expression by brain endothelial cells from infected and naive mice (C). FSC, forward scatter; SSC, side scatter. (D, E) Brain endothelial cells (bEnd5 cell line) were cultured *in vitro* and activated for 18 h with TNF (1 ng/ml) and/or IFN-γ (1 ng/ml). Representative flow cytometry histogram (D) and calculated mean MFI (E) of MHC class I expression by unstimulated and stimulated bEnd5 cells. (F, G) Representative histogram (F) and MFI (G) showing MHC class I expression by brain endothelial cells from P. berghei ANKA-infected and naive mice. (H to J) MFI of VCAM-1 (H) and ICAM-1 (I) on and production of IL-6 (J) by stimulated and unstimulated bEnd5 cells, measured by ELISA. Results in panels C and G are the mean values ± SEM of the groups, with 5 mice per group. Results in panels E and H to J are the mean values ± SEM from three independent biological replicates and are representative of 2 independent experiments. *, *P* < 0.05 between defined groups. Statistical significance was tested using the Mann-Whitney test for the data in panels C and G and one-way ANOVA with Tukey's *post hoc* test for the data in panels E and H to J.

As neuronal (and astrocytic) IFN-γR2 signaling was not required for ECM development, we addressed which other nonhematopoietic cell populations express IFN-γR and may respond to IFN-γ to promote cerebral pathology during P. berghei ANKA infection. Accumulating evidence suggests that brain endothelial cells forming the blood-brain barrier play a pivotal role in ECM pathogenesis through the production of chemokines, cross-presentation of malarial antigens, and regulation of the permeation of materials into the brain tissue (5, 9, 13, 32, 36). Brain Sca-1^+^ CD31^+^ endothelial cells (gated as shown in Fig. 6A, using the strategy defined in reference 37) expressed the IFN-γR subunits in naive mice and during ECM (Fig. 6B and C). Therefore, we hypothesized that endothelial cells were the main nonhematopoietic cell population that responds directly to IFN-γ, leading to the development of ECM. To examine this, we stimulated brain endothelial cells (bEnd5 cells), which also express IFN-γR (results not shown), with IFN-γ, TNF, or both cytokines in combination. IFN-γ promoted significantly higher upregulation of MHC class I expression by bEnd5 cells than TNF (Fig. 6D and E). Indeed, costimulation with IFN-γ and TNF failed to increase MHC class I expression above that induced by IFN-γ by itself (Fig. 6D and E). Of relevance, and as previously reported (32), MHC class I expression by brain endothelial cells was significantly upregulated in WT mice during ECM compared with its expression in naive WT mice (Fig. 6F and G). In contrast, IFN-γ was unable to promote VCAM-1 or ICAM-1 expression or interleukin-6 (IL-6) production by brain endothelial cells by itself *in vitro* and instead only synergized with and amplified the expression and production induced by TNF (Fig. 6H to J). As MHC class I gene expression was significantly lower in the brains of globally IFN-γR2^−/−^ mice and, to a lesser extent, VAV-Cre^+^ IFN-γR2^flox/flox^ mice compared with its expression in WT mice on day 7 of P. berghei ANKA infection (Fig. 3D), these data in combination indicate that IFN-γ may be the dominant (direct and indirect) driver of MHC class I expression by brain endothelial cells during P. berghei ANKA infection. IFN-γ may also play a pathogenic role by synergizing with other inflammatory cytokines to amplify endothelial cell activation, leading to the development of cerebral pathology.

## DISCUSSION

In this study, we have utilized novel cell- and compartment-specific IFN-γR2^−/−^ mice to examine which cell type(s) IFN-γ targets to cause ECM during P. berghei ANKA infection. We found that VAV-Cre^+^ IFN-γR2^flox/flox^ mice displayed intermediate resistance to ECM, with 100% of mice developing prodromal disease and 40% of mice developing late-stage ECM. Thus, we have demonstrated that IFN-γ signals throughout both the hematopoietic and nonhematopoietic compartments to cause ECM.

Notably, abrogation of hematopoietic compartment-specific IFN-γR2 signaling did not significantly modulate the splenic innate or adaptive immune responses during infection. In contrast, hematopoietic and nonhematopoietic IFN-γR2 signaling appeared to function additively to promote malaria-induced cerebral inflammation. As previously reported, IFN-γR2 signaling was critically required for the expression within the brain of a number of chemokine genes (7, 12), as well as genes controlling antigen processing and MHC class I peptide presentation. Unexpectedly, the reduced inflammatory landscape in the brains of infected VAV-Cre^+^ IFN-γR2^flox/flox^ mice was not simply due to reduced CD8^+^ T cell accumulation. Thus, our results are consistent with the model where either redundant global or nonhematopoietic compartment-specific IFN-γR2 signaling recruits CD8^+^ T cells to the brain during P. berghei ANKA infection, most probably through control of chemokine expression in the brain, as the expression of CXCL9 and CXCL10 was reduced but not abrogated in the brains of VAV-Cre^+^ IFN-γR2^flox/flox^ mice. Nonhematopoietic and hematopoietic IFN-γR2 signaling subsequently combine to establish an environment allowing CD8^+^ T cells to optimally mediate their pathogenic activity. Indeed, we have previously shown that the behavior of intracerebral CD8^+^ T cells is as important as their presence *per se* in promoting ECM development (38). Of note, a higher level of brain-to-brain variability in gene expression was observed in the infected VAV-Cre^+^ IFN-γR2^flox/flox^ group than in the infected WT or the IFN-γR2^−/−^ group. This likely reflects the heterogeneity in the susceptibilities of VAV-Cre^+^ IFN-γR2^flox/flox^ mice to ECM and suggests that ECM develops following breaching of threshold levels of expression of inflammation-related genes. Although we cannot fully exclude the possibility that alterations in immune cell development contributed to the partial resistance of VAV-Cre^+^ IFN-γR2^flox/flox^ mice to ECM, the T cell numbers and activation were normal in the spleens and brains of uninfected IFN-γR^−/−^ mice and VAV-Cre^+^ IFN-γR2^flox/flox^ mice (results not shown). Thus, we believe it is unlikely that T cell development was significantly affected by global or hematopoietic compartment-specific abrogation of IFN-γR2 expression.

We dissected the targets of IFN-γ within the hematopoietic lineage and determined that IFN-γR signaling within T cells (CD4-Cre) and monocytes/macrophages and neutrophils (LysM-Cre) is (individually) dispensable for the development of ECM. That IFN-γR expression on T cells is not required for the development of ECM was perhaps unsurprising given that we have previously shown that CD8^+^ T cell activation is largely unimpaired systemically in IFN-γ knockout (KO) mice during P. berghei ANKA infection (11). Moreover, CD4^+^ T cell activation is altered, to a relatively small degree, only in the brains of IFN-γ KO mice during infection (11). Thus, T cell-intrinsic IFN-γR signaling does not appear to contribute to the development of pathogenic T cell responses during P. berghei ANKA infection. Rather, IFN-γ indirectly promotes the migration to and accumulation of CD8^+^ T cells within the brain during P. berghei ANKA infection by modulating the expression of chemokines within the tissue (7, 11, 12).

The observation that LysM-Cre^+^ IFN-γR2^flox/flox^ mice were as susceptible to ECM as WT mice indicates that IFN-γR signaling within blood-derived monocytes, macrophages, and neutrophils is not required for the development of ECM. These results are thus consistent with the more general observations in the literature that peripheral monocytes and macrophages are not critically involved in the pathogenesis of ECM (9, 39, 40). It is important to note, however, that while LysM-Cre has a high gene recombination rate in peripheral monocytes, macrophages, and neutrophils, it only promotes gene recombination in 50% of microglial cells (41). Moreover, the efficiency of VAV-Cre-mediated genetic recombination in microglial cells is debated (42). Consequently, we cannot definitively extrapolate the role of IFN-γR signaling in microglial cells in the development of neuropathology during malaria infection using the Cre-driving lines employed in this study. Nevertheless, utilizing CX3CR1-iDTR mice, in which diphtheria toxin (DT) treatment promotes extensive depletion of microglial cells, perivascular macrophages, and other myeloid cell populations (43), we have recently demonstrated that perivascular macrophages and microglial cells are not involved in the terminal stages of ECM development (38). Consequently, we do not believe that IFN-γR signaling by microglial cells is critically required for ECM development. It remains to be resolved whether IFN-γ acts in a redundant and/or additive manner throughout the hematopoietic compartment to promote ECM or whether it mediates its effects through an as-yet-unidentified hematopoietic cell population.

In terms of the nonhematopoietic cell populations that respond to IFN-γ during P. berghei ANKA infection, our results indicate that neuronal IFN-γR signaling is not crucial for the development of late-stage neuropathology. This suggests that the IFN-γ-dependent effects on neuronal cells observed during ECM (33) must be mediated in *trans* via activity on other cell types. As Nestin-Cre also exerts strong effects in the astrocyte lineage cells (35), our results also suggest that astrocytic IFN-γR signaling is dispensable for the development of ECM.

Notably, it has recently been shown that IFN-γ promotes cross-presentation of malarial antigens by brain endothelial cells and that this is an important event in ECM pathogenesis (13). Consistent with this, we found that IFN-γR was expressed by brain endothelial cells in naive mice and during P. berghei ANKA infection. Moreover, as has previously been reported, we found that IFN-γ stimulation was able to promote strong upregulation of MHC class I by brain endothelial cells *in vitro* (13, 44). In addition, the gene expression of MHC class I was lower in the brains of globally IFN-γR2^−/−^ mice and, to a lesser extent, VAV-Cre^+^ IFN-γR2^flox/flox^ mice than in the brains WT mice on day 7 of P. berghei ANKA infection. As the VAV-Cre line utilized in this study does not promote gene recombination in vascular endothelial cells (45), our results therefore suggest that IFN-γ may nonredundantly directly and indirectly target brain endothelial cells to control MHC class I expression during P. berghei ANKA infection, leading to the development of ECM. Although IFN-γ, in combination with other inflammatory cytokines, also promoted VCAM-1 and ICAM-1 expression and IL-6 production, the gene expression levels of VCAM-1 and ICAM-1 were not significantly lower in the brains of global IFN-γR2^−/−^ mice and VAV-Cre^+^ IFN-γR2^flox/flox^ mice than in the brains of WT mice on day 7 of P. berghei ANKA infection. As it has recently been reported that the protein levels of ICAM-1 and VCAM-1 are reduced by brain endothelial cells in IFN-γ^−/−^ mice during P. berghei ANKA infection (46), this may suggest that there is a dichotomy in the gene and protein expression levels of ICAM-1 and VCAM-1 by brain endothelial cells during P. berghei ANKA infection or that gene expression differences specifically by brain endothelial cells in the IFN-γR2-deficient mice were diluted within our whole-brain NanoString analysis. Thus, although we acknowledge the reductionist nature of our *in vitro* experiments and the fact that they do not fully recapitulate the inflammatory intracerebral environment during ECM, we believe our data, in agreement with previous studies (5, 44), strongly support the hypothesis that brain endothelial cells may be the major nonhematopoietic cell IFN-γ target leading to ECM. In future work, the relative importance of IFN-γ targeting of brain endothelial cells in promoting ECM can be definitively assessed by utilizing Slco1c1-Cre mice (47) crossed with IFN-γR2^flox/flox^ mice.

In summary, the results in this study are consistent with an overall model where IFN-γ acts on different cell populations within the hematopoietic and nonhematopoietic cell compartments to orchestrate brain inflammation that enables CD8^+^ T cells to manifest their pathogenic activity. It remains to be defined whether IFN-γ acts on the diverse responding cell types in a concomitant or temporal manner to promote ECM. However, our results further argue that IFN-γ predominantly exerts its ECM-inducing pathogenic function locally within the brain, rather than within the spleen. The results in this study improve our understanding of the responding cell types that orchestrate the IFN-γ-dependent development of ECM.

## MATERIALS AND METHODS

### Animals and P. berghei ANKA infection.

IFN-γR2^−/−^ (global), VAV-Cre^+/−^ × IFN-γR2^flox/flox^, CD4-Cre^+/−^ × IFN-γR2^flox/flox^, LysM-Cre^+/−^ × IFN-γR2^flox/flox^, and Nestin-Cre^+/−^ × IFN-γR2^flox/flox^ mice (all on the C57BL/6 background) were maintained in individual ventilated cages at the University of Manchester. Within each colony, Cre^+/−^ (heterozygotes with selective IFN-γR2 deficiency) and Cre^−/−^ (WT) mice were identified by PCR. C57BL/6 mice purchased from Charles River, United Kingdom, were used as controls for IFN-γR2^−/−^ mice, and Cre^−^ littermates were used as WT controls for all other strains of mice. The generation and characterization of IFN-γR2^flox/flox^ mice and the efficiency and specificity of the various Cre deletion strains utilized in the experiments have previously been described (31, 35, 48–50). Importantly, as opposed to other VAV-Cre lines (51), the VAV-Cre line utilized in this study does not promote gene recombination in vascular endothelial cells (45). All animal work was approved by the University of Manchester Animal Procedures and Ethics Committee and was performed in accordance with the UK Home Office (HO) Animals (Scientific Procedures) Act 1986 (HO project license 70/7293) (52).

Cryopreserved P. berghei ANKA-GFP (expressing green fluorescent protein) parasites (53) were thawed and passaged once through C57BL/6 mice before being used to infect experimental animals. Animals were infected via intravenous injections of 1 × 10^4^ parasitized red blood cells (pRBCs). Peripheral parasite burdens of infected mice were assessed every day of infection (from day 3 of infection) by microscopic examination of Giemsa-stained thin blood smears. The development of ECM was assessed using a well-established clinical scale (14) as follows: 1, no signs; 2, ruffled fur/and or abnormal posture; 3, lethargy; 4, reduced responsiveness to stimulation and/or ataxia and/or respiratory distress/hyperventilation; and 5, prostration and/or paralysis and/or convulsions. Stages 2 and 3 were classified as prodromal ECM, and stages 4 and 5 were classified as ECM. P. berghei ANKA-infected mice were euthanized when they reached stage 4/5 (typically day 7 of infection).

### Flow cytometry.

Spleens were obtained from infected and uninfected mice. Single-cell suspensions were prepared by homogenizing the tissue through a 70-μm cell strainer (BD Biosciences). Following intracardial perfusion with 10 ml phosphate-buffered saline (PBS), brains were removed from mice, finely chopped, and incubated with 2 mg/ml collagenase D (Sigma) and 28 U/ml DNase for 30 min at 37°C on a tube roller. The suspension was filtered through a 70-μm cell sieve and centrifuged, and the pellet was resuspended in 15 ml 37% Percoll solution and centrifuged at 2,000 × *g* for 10 min. The myelin layer was removed, and the sample was washed in fluorescence-activated cell sorting (FACS) buffer (Hanks balanced salt solution [HBSS] with 2% fetal calf serum [FCS]). Red blood cells (RBCs) were lysed in spleen and brain samples by the addition of BD RBC lyse (BD Biosciences), and the samples were washed and resuspended in FACS buffer. The samples were then surface stained with anti-mouse antibodies against CD4 (GK1.5), CD8 (53-6.7), CD11b (M1/70), Ly6C (HK1.4), Ly6G (RB6.8C5), CD11c (N418), CD3 (17A2), F4-80 (BM8), ICOS (C398.4A), KLRG-1 (2F1), CD40 (1C10), CD31 (390), Sca-1 (D7), CD45 (30-F11), and MHC class II (M5/114.15.2). For intracellular staining, surface-stained cells were washed in FACS buffer and permeabilized with Foxp3 fixation/permeabilization buffers (eBioscience) for 30 min. The cells were then stained with anti-mouse antibodies against Ki-67 (16A8) and granzyme B (NGZB) for 30 min. All antibodies were from eBioscience, Biolegend, or R&D Systems. Fluorescence-minus-one (FMO) controls were performed to validate antibody staining. Data acquisition was performed using an LSR II instrument (BD Systems, United Kingdom), and analysis was performed using FlowJo software (Tree Star, Inc., Ashland, OR, USA).

### NanoString.

Brains were removed from infected and uninfected mice following intracardiac perfusion with PBS. The brains were bisected, snap-frozen in liquid N_2_, and stored at −80°C until required. RNA was extracted from the half brains using the RNeasy lipid tissue kit (Qiagen) according to the manufacturer's guidelines. The nCounter gene expression assay (NanoString Technologies, Seattle, WA, USA) was performed according to the manufacturer's instructions. Briefly, 100 ng of RNA was hybridized with the reporter and capture probe sets at 65°C overnight. Unhybridized probes were removed via an automated purification performed on the nCounter preparation station, and the resulting target-probe complexes were deposited and bound to the imaging surface. Voltage was applied to elongate and align the molecules, which were then immobilized for imaging. Images were acquired using the nCounter digital analyzer, up to a maximum resolution of 555 fields of view (FOV) per sample. Transcript counts were normalized to the transcription of relevant housekeeping genes using nSolver Analysis software (version 2.5; NanoString Technologies).

### *In vitro* activation of brain endothelial cells.

Cells from the murine brain endothelial cell line bEnd5 were seeded in a 24-well plate at 0.1 × 10^6^ cell density/well and stimulated for 24 h with 1 ng/ml IFN-γ and/or 1 ng/ml TNF. Cell activation was measured by flow cytometry, as described above. Cells were detached using StemPro Accutase cell dissociation reagent (Thermo Fisher Scientific). The samples were stained with the fixable viability dye eFluor 780 (eBioscience), followed by surface staining with anti-mouse antibodies against CD106 (VCAM-I; clone 429), CD119 (IFN-γR1; clone 2E2), IFN-γR2 (MOB-47), MHC class I (H-2K^b^/H-2D^b^; clone 28-8-6), and CD54 (ICAM-I; clone YN1/1.7.4). All antibodies were from eBioscience or Biolegend.

### ELISA.

The expression of IL-6 by bEnd5 cells activated *in vitro* by IFN-γ and/or TNF was measured from cell supernatants using an IL-6 enzyme-linked immunosorbent assay (ELISA) (R&D systems), following the manufacturer's instructions. Optical densities were read in a plate reader at 450 nm, with correction for baseline at 570 nm (Synergy HT plate reader; BioTek). The concentrations of the samples were determined by interpolating from a standard curve fitted by sigmoidal equation.

### Statistical analysis.

Data were checked for normality using the Shapiro-Wilk test. For two-group comparisons, statistical significance was determined using the *t* test (parametric data) or Mann-Whitney test (nonparametric data). For comparisons of three or more groups, statistical significance was determined using one-way or two-way analysis of variance (ANOVA) with Tukey *post hoc* analysis (parametric data) or the Kruskal-Wallis test with Dunn's *post hoc* test (nonparametric data). Results were considered significantly different when the *P* value was <0.05.
